# Supplementary material for: Association of predicted lean body mass and fat mass with prognosis in patients with heart failure preserved ejection fraction
Source: PLoS One. 2025 May 30;20(5):e0323634. doi: 10.1371/journal.pone.0323634 (PMC12124579; doi:10.1371/journal.pone.0323634)
Supplement: S1 File — S1 Table. Prediction equations for Lean BMI, FMI and percent fat. S2 Table. Effects of spironolactone on body composition in different subgroups. S3 Table. Hazard ratio (95% CI) of Primary endpoint according to fifths of Lean BMI and FMI. S4 Table. Hazard ratio (95% CI) of All cause death according to fifths of Lean BMI and FMI. S5 Table. Hazard ratio (95% CI) of Primary endpoint according to thirds of Lean BMI and FMI. S6 Table. Hazard ratio (95% CI) of All cause death according to thirds of Lean BMI and FMI. S7 Table. Sensitivity analysis of Lean BMI and FMI in relation to Primary endpoint by excluding Primary endpoint occurred early in the first two years. S8 Table. Sensitivity analysis of Lean BMI and FMI in relation to All cause death by excluding All cause death occurred early in the first two years. S9 Table. Sensitivity analysis of Lean BMI and FMI in relation to Primary endpoint by excluding patients with age > 70 years. S10 Table. Sensitivity analysis of Lean BMI and FMI in relation to All cause death by excluding patients with age > 70 years. (ZIP) [file pone.0323634.s001.zip › Supporting information/S3_table.docx]

| **[S3. Table1: Hazard ratio (95% CI) of Primary endpoint according to fifths of Lean](https://www.cmaj.ca/highwire/filestream/73705/field_highwire_adjunct_files/3/190124-res-3-at.pdf) BMI** | | | | |
| --- | --- | --- | --- | --- |
| Lean BMI fifths | HR (95%CI) | | | |
|  | model1* | model2† | model3‡ | model4§ |
| 1st | Ref. | Ref. | Ref. | Ref. |
| 2nd | 0.65(0.49-0.85) | 0.72(0.55-0.95) | 0.70(0.53-0.92) | 0.66(0.50-0.87) |
| 3rd | 0.81(0.63-1.05) | 0.96(0.75-1.24) | 0.88(0.68-1.14) | 0.78(0.59-1.03) |
| 4th | 0.91(0.71-1.17) | 1.07(0.83-1.37) | 0.87(0.67-1.13) | 0.72(0.53-0.98) |
| 5th | 1.49(1.19-1.88) | 2.01(1.58-2.55) | 1.45(1.13-1.88) | 1.03(0.69-1.52) |
| trend P | 0.30 | 0.23 | 0.26 | 0.26 |
| [**S3. Table2: Hazard ratio (95% CI) of Primary endpoint according to fifths of FMI**](https://www.cmaj.ca/highwire/filestream/73705/field_highwire_adjunct_files/3/190124-res-3-at.pdf) | | | | |
| FMI fifths | HR (95%CI) | | | |
|  | model1* | model2† | model3‡ | model4§ |
| 1st | Ref. | Ref. | Ref. | Ref. |
| 2nd | 0.78(0.60-1.03) | 0.81(0.62-1.07) | 0.78(0.60-1.03) | 0.77(0.58-1.01) |
| 3rd | 0.99(0.76-1.28) | 1.02(0.78-1.32) | 0.89(0.68-1.16) | 0.85(0.65-1.13) |
| 4th | 1.18(0.91-1.52) | 1.26(0.98-1.63) | 1.02(0.79-1.33) | 0.95(0.71-1.28) |
| 5th | 1.75(1.38-2.21) | 2.00(1.58-2.55) | 1.44(1.12-1.86) | 1.27(0.88-1.82) |
| trend P | 0.12 | 0.13 | 0.15 | 0.16 |

Note: BMI = body mass index, FMI = Fat mass index, CI = confidence interval, HR = hazard ratio, Ref. = reference.

*Unadjusted.

†Adjusted for age, sex and race.

‡Adjusted for age, sex, race, spironolactone treatment, history of CAD, hyperlipidemia, hypertension, smoking, diabetes, and COPD

§Adjusted using characteristics for Model 3 by adding Fat mass index or Lean BMI.
