# Supplementary material for: Association of predicted lean body mass and fat mass with prognosis in patients with heart failure preserved ejection fraction
Source: PLoS One. 2025 May 30;20(5):e0323634. doi: 10.1371/journal.pone.0323634 (PMC12124579; doi:10.1371/journal.pone.0323634)
Supplement: Attachment — Submitted filename: Response to Reviewers.docx [file pone.0323634.s003.docx]

Response to Reviewers

Dear Reviewers:

Thank you for reviewing our study. Your comments are very valuable and have greatly helped us improve the content of the paper. Now I will respond to your suggestions for improvement and hope to get your positive response.

1. I suggest to expand the discussion section including more previous studies and trying to suggest more clinical implication. Moreover, could you analyze the impact of spironolactone in different lean BMI/FMI subgroups?

Thanks to your suggestions, we have supplemented the discussion section. We believe that the use of Lean BMI and FMI to evaluate the prognosis of HFpEF patients has positive significance. At the same time, these two indicators provide guidance for guiding patients to conduct exercise rehabilitation and body composition adjustment. At the same time, according to your suggestions, we have improved the analysis of the impact of spironolactone in Lean BMI and FMI for different subgroups, and the results are shown as supplementary material S2 Table. At the same time, we supplemented the use data of spironolactone in the study population in Table 1. I hope these contents can meet your expectations.

2. Several studies have shown that it is the waist circumference (and not BMI) that is a significant risk factor for many cardiovascular disorders and cardiovascular events. I believe at least some of these studies must be discussed in your manuscript since visceral fat, lean body mass, and fat mass are greatly emphasizing the waist circumference over BMI. Here are some articles that I recommend to add to your manuscript: PMID: 32020062 (discusses importance of waist circumference) ,PMID: 37227560 ( shows significant association between major cardiovascular events including heart failure and waist circumference, I highly recommend both)

Thank you for your suggestion. Waist circumference is indeed an important risk factor for many cardiovascular diseases and cardiovascular events. To this end, we show the effect of waist circumference on the primary endpoint and all cuase death of patients in Table 2. As you said, waist circumference is a very important factor. Lean BMI and FMI include waist circumference as an important indicator in the calculation process, which can also partially reflect the important position of waist circumference in our study. Regarding the reference PMID: 32020062 you recommended, we appreciate your help and have included it in our references, which is very helpful for our discussion. Regarding the reference PMID: 37227560, unfortunately, we read this article and found it not relevant to our research, so we did not include it. However, your caption inspired us, and we included more content mentioned in your caption and discussed it, and also added relevant references. Thank you very much for your suggestions, and I hope these contents can meet your expectations.

Junyu Pei and Wen Su
